# The Correlation and Clinicopathological Significance of TNFAIP8L3 and RAC1 Expression in Lung Adenocarcinoma

**DOI:** 10.1155/genr/9994311

**Published:** 2025-08-26

**Authors:** Xuexin Shi, Haitao Guo, Kaihua Tian

**Affiliations:** ^1^Department of Thoracic Surgery, Qingdao Municipal Hospital, Qingdao 266000, Shandong, China; ^2^Department of Thoracic Surgery, Shandong Provincial Public Health Clinical Center Qingdao Branch, Qingdao Sixth People's Hospital, Qingdao 266000, Shandong, China; ^3^Department of Thoracic Surgery, The Affiliated Hospital of Qingdao University, Qingdao 266000, Shandong, China

**Keywords:** adenocarcinoma of the lung, biomarkers, prognosis, RAC1, survival analysis, TNFAIP8L3

## Abstract

**Background:** Lung adenocarcinoma (LUAD) remains one of the leading causes of cancer-related mortality worldwide. However, the expression and role of TIPE3 and RAC1 in LUAD are not well characterized.

**Objective:** This study aimed to investigate the expression and clinicopathological significance of TNFAIP8L3 (TIPE3) and RAC1 in LUAD, as well as the relationship between these two proteins.

**Methods:** Immunohistochemistry (IHC) was utilized to detect the expression of TIPE3 and RAC1 in tumor and adjacent normal tissues from 183 LUAD patients. A comprehensive analysis of clinicopathological data and subsequent follow-up outcomes was conducted in relation to TIPE3 and RAC1 expression levels. The correlation between these two proteins was also evaluated.

**Results:** Both TIPE3 and RAC1 expression were upregulated in tumor tissues of LUAD. TIPE3 expression was significantly associated with advanced T stage (*p*=0.001), N stage (*p*=0.005), and TNM stage (*p*=0.001). Similarly, increased RAC1 expression was also associated with advanced T stage (*p*=0.003), N stage (*p*=0.003), and TNM stage (*p*=0.001). Kaplan–Meier survival analysis and Cox regression modeling demonstrated that increased TIPE3 and RAC1 expression were independent prognostic factors for poor outcomes in LUAD. Furthermore, Spearman correlation analysis revealed a positive association between TIPE3 and RAC1 expression (*r* = 0.305, *p* < 0.001). Combined expression of TIPE3 and RAC1 improved risk stratification and prognostic prediction in LUAD.

**Conclusion:** TIPE3 and RAC1 serve as potential biomarkers of tumor progression and poor prognosis in LUAD, offering promising targets for future therapeutic interventions.

## 1. Introduction

Lung cancer remains the most common malignancy and the leading cause of cancer-related deaths globally [1, 2]. The major histological types of lung cancer include lung adenocarcinoma (LUAD), squamous carcinoma, adeno-squamous carcinoma, large cell carcinoma, and small cell carcinoma [3]. LUAD is the most prevalent subtype, leading to a heavy burden on human health and social development. The predominant treatment strategy for LUAD involves a combination of surgical resection, chemotherapy, and targeted therapy. Despite clinical progression in the diagnosis and systematic treatment of LUAD in the recent decades, the long-term survival of LUAD patients remains unsatisfactory [4, 5]. Consequently, the identification of novel biomarkers and the delineation of potential therapeutic targets are imperative for enhancing the prognosis of LUAD patients [6].

The tumor necrosis factor-alpha-induced protein 8 (TNFAIP8 or TIPE) family consists of four members: TNFAIP8 (TIPE), TNFAIP8L1 (TIPE1), TNFAIP8L2 (TIPE2), and TNFAIP8L3 (TIPE3). These proteins play a critical role in the regulation of tumorigenesis and inflammation [7–9]. TIPE3 is the most recently described member of the TIPE family and exhibits high structural homology with the other TIPE family members. The aberrant expression of TIPE3 has been identified in a variety of neoplasms, including nasopharyngeal cancer, gastric carcinoma, glioblastoma, and lung cancer [10–16]. TIPE3 primarily functions as a tumor promoter during the processes of tumorigenesis and tumor progression. However, the detailed expression of TIPE3 protein in LUAD and its related clinicopathological significance are largely unknown.

RAC1 is classified as a member of the Ras superfamily, which encompasses a group of small GTPases. It has been established that RAC1 plays a pivotal role in a variety of important cellular processes, including gene transcription, cell adhesion, cell movement, and cell cycle progression [17, 18]. Aberrant RAC1 activity has been implicated in tumorigenesis [19, 20]. Previous studies have demonstrated that members of the TIPE family may influence tumor progression through interactions with RAC1. For example, TIPE2 suppresses tumor growth by directly binding to RAC1, whereas TIPE3 promotes pancreatic cancer progression in a RAC1-dependent manner. Furthermore, there is evidence to suggest that RAC1 plays a role in the progression of lung cancer [21, 22].

In the present study, we established a retrospective cohort composed of 183 LUAD patients to explore the clinicopathological significance of TIPE3 and RAC1 in LUAD. The study identified a potential target for risk stratification and prognostic prediction in patients with LUAD.

## 2. Patients and Methods

### 2.1. Patients and Data Collection

This retrospective cohort study included 183 patients diagnosed with LUAD who underwent surgical resections with R0 margins at the Department of Thoracic Surgery, Affiliated Hospital of Qingdao University, between 2013 and 2016. The diagnosis of LUAD was confirmed through routine pathological examination, and the inclusion criteria were as follows:

1. Formalin-fixed tumor tissues and corresponding adjacent normal tissues available, accompanied by comprehensive medical records.
2. No prior adjuvant chemotherapy or radiotherapy.
3. Postoperative survival exceeding 1 month (to exclude death from postoperative complications or comorbidities, which may interfere the survival results).
4. No history or clinical evidence of other malignancies.

Follow-up data, collected until December 2021, provided survival information for the last visit. Tumor staging and histological classifications were determined according to the eighth edition of the American Joint Committee on Cancer (AJCC) classification. The study was approved by the Ethics Committee of the Affiliated Hospital of Qingdao University, China (approval number: QYFYWZLL29111). Written informed consent was obtained from all patients. All procedures involving human subjects complied with the ethical principles outlined in the Declaration of Helsinki.

### 2.2. Immunohistochemistry (IHC)

IHC analysis was performed on paraffin-embedded tissue sections. The sections were deparaffinized and hydrated, followed by antigen retrieval using a 0.01 mol/L citrate buffer solution (pH 6.0) heated to boiling for 2–3 min. The endogenous peroxidase activity was blocked using 3% H_2_O_2_. Subsequently, the sections were incubated with blocking goat serum for 15 min and then subjected to overnight immunostaining at 4°C with rabbit antibody against TIPE3 or mouse antibody against RAC1 at 4°C overnight [TIPE3 antibody, dilution 1:300, BOSTER, China (11). RAC1 antibody, dilution 1:300, Abcam (ab97732), UK]. Secondary staining was performed with HRP-conjugated antirabbit or antimouse IgG using a MaxVision Kit and a 3, 5-diaminobenzidine (DAB) peroxidase substrate kit (Maixin Co., Fuzhou, China). The slides were subsequently counterstained with hematoxylin, and representative images were obtained under an Olympus inverted microscope.

### 2.3. Assessment of Immunohistochemical Staining

The assessment of immunohistochemical staining was conducted independently by two experienced pathologists who performed the analysis in a blinded manner. Staining intensity was semiquantitatively graded according to both the staining intensity (0, negative; 1, very weak; 2, weak; 3, moderate; 4, strong) and the percentage of positively stained cells (0, 0%; 1, 1%–25%; 2, 26%–50%; 3, 51%–75%; 4, 76%–100%). The staining intensity and percentage scores were summed to calculate the final TIPE3 or RAC1 expression score, ranging from 0 to 8. Expression levels were categorized as follows: 0–4, low expression; 5–8, high expression. To reduce the interobserver agreement, a joint review was performed using a double-headed microscope to reach a consensus and ensure scoring consistency in case of disagreement. The cut-off values were validated using the X-tile program.

### 2.4. Statistical Analysis

All statistical analyses were conducted using SPSS 22.0 software (SPSS, Chicago, IL, USA). Associations between TIPE3/RAC1 expression and clinicopathological parameters were analyzed using the chi-square test or Fisher's exact test. Bonferroni correction was used for adjustment of multiple comparisons. The predictive accuracy index was evaluated through receiver operating characteristic (ROC) curve analysis. Overall survival (OS) rates were calculated using the Kaplan–Meier method, with statistical differences between subgroups determined by the log-rank test. Multivariate analysis using a Cox proportional hazards regression model was conducted to identify independent prognostic factors. A *p* value < 0.05 was considered statistically significant.

## 3. Results

### 3.1. Clinical Characteristics of the Retrospective Cohort

As shown in Table 1, the baseline clinical characteristics of 183 patients diagnosed with LUAD. Among the subjects in this study, 98 (53.5%) were male and 85 (46.5%) were female, with ages ranging from 30 to 84 years. The pathological tumor-node-metastasis (TNM) classification and cancer stage were determined according to the eighth edition of the AJCC stage groupings. A subsequent follow-up was conducted on all patients, and the median survival period was found to be 47.4 months (ranging from 2 to 121 months).

### 3.2. The Expression of TIPE3 and RAC1 in LUAD Cancer Tissues and Adjacent Normal Tissues

**Figure 1:**
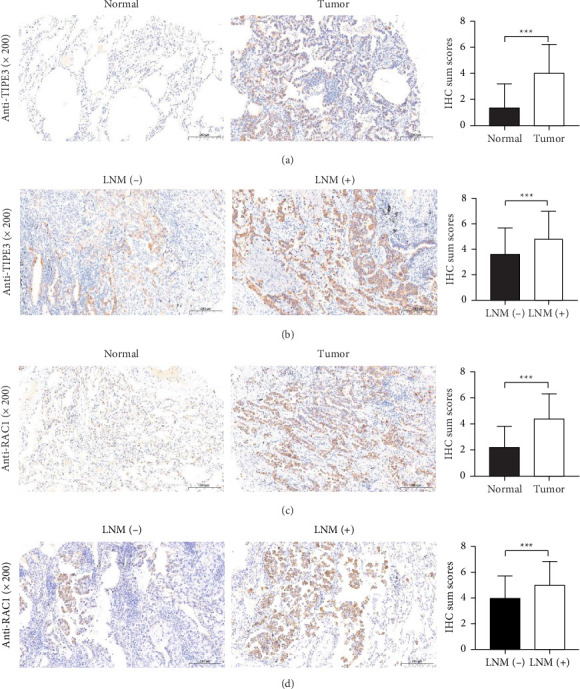
The expression of TIPE3 and RAC1 in tumor tissues and adjacent normal tissues of LUAD patients. (a) The expression of TIPE3 in LUAD tumor tissues and corresponding adjacent normal tissues. (b) The expression of TIPE3 in LUAD tumor tissues with or without lymph node metastasis. (c) The expression of RAC1 in LUAD tumor tissues and corresponding adjacent normal tissues. (d) The expression of RAC1 in LUAD tumor tissues with or without lymph node metastasis. ^∗∗∗^, *p* < 0.001.

To investigate the expression condition of TIPE3 and RAC1 in LUAD, IHC was conducted and as shown in Figure 1, both the expression levels of TIPE3 and RAC1 were significantly elevated in LUAD cancer tissues compared with adjacent normal tissues. TIPE3 is predominantly found in the cytoplasm and membrane of tumor cells, while RAC1 is primarily located in the cytoplasm of tumor cells.

### 3.3. TIPE3 and RAC1 Expression Were Associated With Tumor Stage in LUAD

As demonstrated in Table 1, increased TIPE3 expression exhibited a strong correlation with the T stage (*p*=0.001), N stage (*p*=0.005), and TNM stage (*p*=0.001). Similarly, increased RAC1 expression was also associated with advanced T stage (*p*=0.003), N stage (*p*=0.003), and TNM stage (*p*=0.001). Importantly, both TIPE3 expression and RAC1 expression were significantly increased in tumor tissues of patients with lymph node metastasis (Figure 1). The aforementioned results indicated that TIPE3 and RAC1 may serve as a potential biomarker for LUAD.

### 3.4. High TIPE3/RAC1 Expression Was Associated With Poor Survival of LUAD Patients

**Figure 2:**
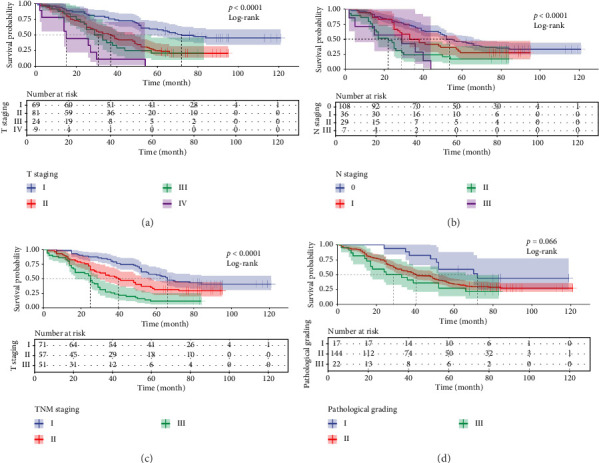
The TNM stage was associated with prognosis of patients with LUAD. (a) Survival analysis based on tumor T stage for LUAD patients. (b) Kaplan-Meier survival curves according to tumor N stage for LUAD patients. (c) Survival analysis based on the TNM stage for patients with LUAD. (d) Kaplan-Meier survival curves according to the pathological grading for patients with LUAD.

**Figure 3:**
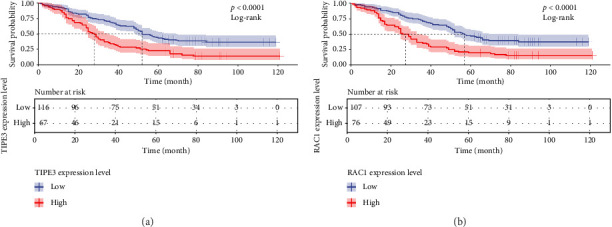
TIPE3 and RAC1 expression may serve as unfavorable prognostic biomarker for LUAD patients. (a) Survival analysis based on tumor TIPE3 expression for patients with LUAD. (b) Kaplan-Meier survival curves according to RAC1 expression for LUAD patients.

To determine the prognostic value of TIPE3 and RAC1 expression in OS of LUAD patients, the Kaplan–Meier analysis was conducted. In this retrospective cohort, patients with advanced T stage (*p*=0.001), N stage (*p*=0.001), TNM stage (*p*=0.001), and advanced pathological grading (*p*=0.007) exhibited a reduced OS (Table 2, Figure 2). Importantly, results found that the OS of LUAD patients with high TIPE3 expression was significantly lower than that of patients with low TIPE3 expression (*p*=0.001). Conversely, patients with high RAC1 expression also showed poor prognosis compared to those with low RAC1 expression (Table 2, Figure 3).

Multivariate analysis was further conducted to evaluate the independent prognostic factors within this cohort. Factors with *p* value ≤ 0.10 in the previous univariate analysis were incorporated into the Cox regression model for the multivariate analysis. The findings indicated that, in addition to advanced T stage (*p*=0.044) and TNM stage (*p*=0.055), high expression of TIPE3 (*p*=0.050) and RAC1 (*p*=0.007) were identified as independent unfavorable prognostic factors (Table 2).

### 3.5. Combined TIPE3 and RAC1 Expression Was Useful for Risk Stratification and Prognostic Prediction in LUAD

As both TIPE3 and RAC1 expression were elevated in LUAD, then we explored the relationship between TIPE3 and RAC1 expression. A subsequent Spearman correlation analysis revealed a positive association between TIPE3 and RAC1 expression (*r* = 0.305, *p* < 0.001, Table 3).

**Figure 4:**
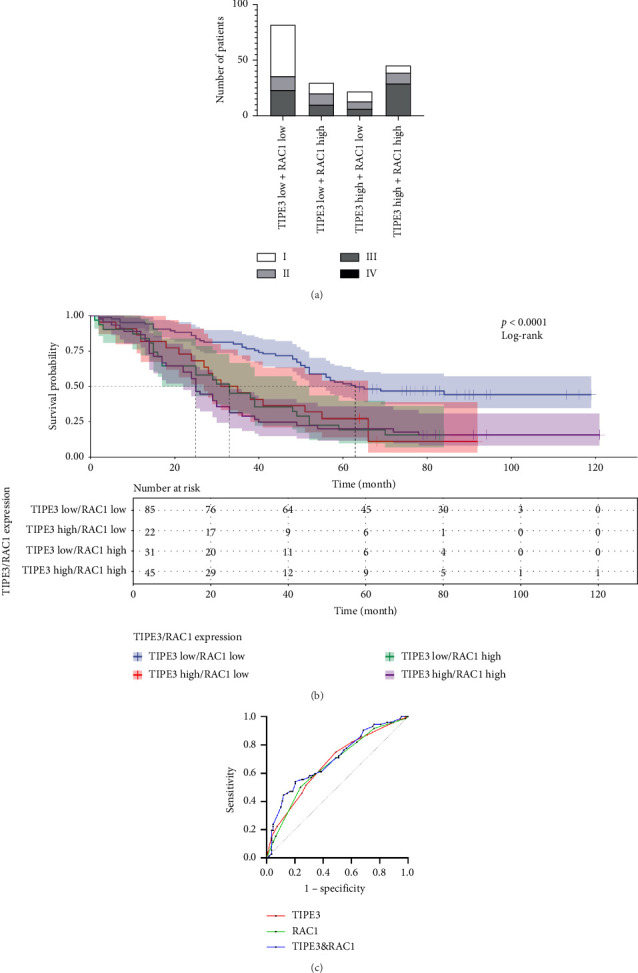
Combined TIPE3 and RAC1 expression was useful for risk stratification and prognostic prediction in LUAD. (a) Different tumor stage proportions for LUAD patients with different TIPE3 and RAC1 expression conditions. (b) Kaplan-Meier survival curves for LUAD patients with different TIPE3 and RAC1 expression conditions. (c) ROC curves for LUAD patients with different TIPE3 and RAC1 expression conditions.

Then, we divided the retrospective cohort into four groups based on the expression of TIPE3 and RAC1, TIPE3 high expression and RAC1 high expression, TIPE3 high expression and RAC1 low expression, TIPE3 low expression and RAC1 high expression, and TIPE3 low expression and RAC1 low expression. Results showed that patients with high TIPE3 and RAC1 expression exhibited a significantly advanced TNM stage, and patients with low TIPE3 and RAC1 expression showed a relatively earlier TNM stage (Figure 4(a)). Of particular significance is the observation that patients exhibiting elevated TIPE3 and RAC1 expression levels demonstrated a significantly diminished OS when compared to the other group (Figure 4(b)). Furthermore, ROC analysis also demonstrated that combined TIPE3 and RAC1 expression effectively predicted poor prognosis of LUAD patients (Figure 4(c)). These results indicated that combined TIPE3 and RAC1 expression may serve as potential biomarkers for risk stratification and prognostic prediction in LUAD.

## 4. Discussion

LUAD remains a significant public health challenge due to its high incidence and mortality. Currently, common IHC markers for LUAD include thyroid transcription factor-1 (TTF-1), Napsin A, and cytokeratin-7 (CK7), but none of these markers has both high sensitivity and specificity. Identifying reliable biomarkers for prognosis and therapeutic targets is critical to improving clinical outcomes [23].

Accumulating evidence has demonstrated the vital importance of TIPE3 in various biological processes, especially in the development and progression of cancer. TIPE3 expression has been found to be significantly elevated in a variety of tumor cells and tissues. This finding suggests that TIPE3 may function as an oncogene [11, 21]. It has been reported that TIPE3 could accelerate the metastasis of breast cancer by activating AKT and NF-kB signaling pathways [24]. While TIPE3 promotes the proliferation of glioblastoma cells via inhibiting p38/MAPK signaling [13, 16]. Previous research also found that membrane-located TIPE3 promotes the proliferation and metastasis of NSCLC cells, and the Wnt signaling may contribute to TIPE3-induced tumor progression [15, 25]. All these aforementioned results demonstrated that TIPE3, the most recently identified member of the TIPE family, acts as an oncogenic molecule that promotes cancer development and progression. However, the detailed expression of TIPE3 and its role in the prognosis of LUAD patients is largely unknown. By constructing a relatively large cohort of LUAD patients, we investigated the clinical significance of TIPE3 expression in LUAD. Consistent with the previous research, the present study found that TIPE3 expression was significantly increased in LUAD tumor tissues compared with adjacent normal tissues. Moreover, we demonstrated that TIPE3 expression is associated with LUAD metastasis, indicating that TIPE3 plays a key role in the progression of LUAD. Furthermore, the expression of TIPE3 could serve as a prognostic factor for metastatic and prognostic prediction in LUAD patients. The results of this study are of paramount importance, as they serve as a crucial addition to the existing body of knowledge concerning the expression and functions of TIPE3. This contributes to the expansion of the theoretical framework underpinning the progression of LUAD. Consequently, to assess the risk and prognosis of patients with LUAD, it is recommended that a standard TIPE3 detection be performed on LUAD tumor tissues.

Previous research found that TIPE3 may promote tumor progression via increasing the expression of RAC1, while other TIPE family members also interact with RAC1 directly during tumor progression (21). RAC1 is an important member of the Rho GTPase family, and it participates in both cell proliferation and tumor metastasis [26, 27]. The hallmarks of cancer include abnormal cell proliferation and metastasis. Consequently, RAC1 has been identified as a critical factor in the initiation and progression of tumors. Furthermore, the present study found that RAC1 was highly expressed in most cancers and closely associated with tumor pathological stages [28]. It has been reported that significant associations were observed between RAC1 and DNA methylation, immune cell infiltration, immune-related genes, tumor mutational burden, and microsatellite instability in most tumors [19, 29, 30]. It is evident that the aforementioned characteristics render RAC1 a promising candidate for targeting by immunotherapy and a valuable prognostic biomarker. A genetic mutation profiling study has identified RAC1 as a potential biomarker for evaluating the efficacy of targeted therapy and for prognostic purposes in NSCLC. While in vitro studies demonstrated downregulation of RAC1 significantly suppressed the migration and invasion of NSCLC cells [31–33]. Here we also explored the detailed expression situation of RAC1 in LUAD. Results also supported that RAC1 is up-regulated in LUAD tumor tissues compared with normal tissues. Increased RAC1 expression was associated with tumor metastasis and the prognosis of LUAD patients. As a commonly used IHC marker, TTF-1 is usually expressed in LUAD, and its expression may decrease accompanied with disease progression, while Napsin A is expressed in approximately 70%–90% of LUAD tissues, but it is also expressed in a variety of tumors such as renal cancer, ovarian cancer, cholangiocarcinoma, etc. CK7 has a high sensitivity but relatively low specificity. These common markers should be combined and used during clinical application. The present study also identified two potential markers for the risk stratification and prognostic prediction of LUAD, but the combined expression seems to have a better efficacy. Moreover, the combination of TIPE3 and RAC1 expression exhibited an optimal value in predicting tumor malignancy. Importantly, we can see a correlation between TIPE3 expression and RAC1 expression in LUAD. It has been reported that TIPE3 may promote tumor progression via increasing RAC1 expression in pancreatic cancer. All these results indicated that the TIPE3 and RAC1 promote tumor progression synergistically. Further investigation is warranted to elucidate the related effects and detailed mechanisms.

Notably, prior studies have identified a relationship between TIPE2, another member of the TIPE family, and RAC1. Different from the oncogenic role of TIPE3, TIPE2 predominantly functions as a tumor suppressor in various types of cancers. Mechanistically, TIPE2 has the capacity to bind directly with RAC1, thereby suppressing its activity and, consequently, hindering tumor progression [32, 34]. As all the members of TIPE family were of high homology, the role and detailed mechanisms behind their effects remains unknown, which require further exploration [8, 35]. Due to the retrospective property of the present study, some potential confounders including smoking history, comorbidities, or treatment regimens were not recorded. Moreover, the single-center design and relatively small cohort size may limit the generalizability of our findings. Multicenter studies with larger cohorts are needed to validate these results and further investigate the therapeutic potential of targeting TIPE3 and RAC1 in LUAD. Practical challenges such as standardization of IHC staining and scoring across institutions should be taken into consideration. Expression of TIPE3 and RAC1 in the blood samples of LUAD patients should also be explored, as blood samples could be easily accessed compared with tumor tissues. Furthermore, the present study identified two potential biomarkers for LUAD, but functional studies and detailed mechanisms beyond their expression properties warrant further investigation.

In conclusion, for the first time, we have demonstrated that both TIPE3 and RAC1 expression levels were elevated in LUAD. TIPE3 and RAC1 have been identified as promising biomarkers for risk stratification and prognostic prediction of LUAD using a retrospective cohort. Moreover, the combined expression of TIPE3 and RAC1 exhibited a favorable prognostic value. The findings of this study yielded novel biomarkers and potential therapeutic targets for the diagnosis and treatment of LUAD.

## Figures and Tables

**Table 1:** The correlation of TIPE3 expression and RAC1 expression with clinicopathologic variables in patients of LUAD.

Characteristic	Number	TIPE3 expression		*χ* ^2^/*t* value	*p* value^∗^	RAC1 expression		*χ* ^2^/*t* value	*p* value^∗^
		Low (%)	High (%)			Low (%)	High (%)		
Age (years)				2.953	1.000			3.926	0.742
< 65	121	82 (44.8)	39 (21.3)			77 (42.1)	44 (24.1)		
≥ 65	62	34 (18.5)	28 (15.3)			30 (16.3)	32 (17.5)		
Tumor location				2.173	1.000			1.416	1.000
Left	68	39 (21.3)	29 (15.8)			40 (21.8)	28 (15.3)		
Right	114	76 (41.5)	38 (20.7)			67 (36.6)	47 (25.6)		
T stage				21.53	0.014			13.508	0.042
T1	69	51 (27.8)	18 (9.8)			50 (27.3)	19 (10.3)		
T2	81	55 (30.0)	26 (14.2)			45 (24.5)	36 (19.6)		
T3	24	9 (4.9)	15 (8.1)			10 (5.4)	14 (7.6)		
T4	9	1 (0.5)	8 (4.3)			2 (1.0)	7 (3.8)		
N stage				12.43	0.070			13.115	0.042
N0	108	78 (43.3)	30 (16.6)			74 (41.1)	34 (18.8)		
N1	36	20 (11.1)	16 (8.8)			18 (10.0)	18 (10.0)		
N2	29	13 (7.2)	16 (8.8)			10 (5.5)	19 (10.5)		
N3	7	2 (1.1)	5 (2.7)			3 (1.6)	4 (2.2)		
TNM stage				22.14	0.014			23.877	0.014
I	71	56 (31.2)	15 (8.3)			55 (30.7)	16 (8.9)		
II	57	37 (20.6)	20 (11.1)			32 (17.8)	25 (13.9)		
III	51	19 (10.6)	32 (17.8)			17 (9.4)	34 (18.9)		
Expression in normal tissues				0.414	1.000			0.259	1.000
Negative	104	68 (37.1)	36 (19.6)			26 (14.2)	21 (11.4)		
Positive	79	48 (26.2)	31 (16.9)			81 (44.2)	55 (30.0)		
Pathological grading				4.391	1.000			4.425	1.000
I	17	13 (7.1)	4 (2.1)			14 (7.6)	3 (1.6)		
II	144	93 (50.8)	51 (27.8)			81 (44.2)	63 (34.4)		
III	22	10 (5.4)	12 (6.5)			12 (6.5)	10 (5.4)		

*Note:* Significance: ^∗^*p* < 0.05, ^∗∗^*p* < 0.01 (after correction).

^∗^All *p* values were adjusted for multiple comparisons using the Bonferroni correction.

**Table 2:** Univariate and multivariate analyses applying the Cox proportional hazard model to patients diagnosed with LUAD.

Variable	Univariate analysis		Multivariate analysis	
	HR (95% CI)	*p-*value	HR (95% CI)	*p* value
Sex	1.246 (0.881–1.763)	0.214		
T stage		0.001		0.044
T1	Reference		Reference	
T2	2.233 (1.483–3.362)	0.001	1.804 (1.121–2.902)	0.015
T3	2.517 (1.439–4.404)	0.001	1.385 (0.687–2.792)	0.362
T4	6.043 (2.863–12.755)	0.001	2.595 (1.066–6.318)	0.036
N stage		0.001		
N0	Reference			
N1	1.538 (0.966–2.449)	0.700		
N2	2.766 (1.708–4.479)	0.001		
N3	4.014 (1.806–8.922)	0.001		
TNM stage		0.001		0.055
I	Reference		Reference	
II	1.512 (0.934–2.447)	0.092	1.277 (0.784–2.081)	0.326
III	3.160 (2.096–4.765)	0.001	2.028 (1.127–3.648)	0.018
Pathological grading		0.007		
I	Reference			
II	1.901 (0.961–3.759)	0.065		
III	2.543 (1.132–5.713)	0.024		
TIPE3 expression level	1.230 (1.133–1.336)	0.001		0.050
RAC1 expression level	1.318 (1.193–1.456)	0.001	1.695 (1.158–2.480)	0.007

Abbreviations: CI, confidence interval; HR, hazard ratio.

**Table 3:** The correlation between the expression of TIPE3 and RAC1 in tumor tissues.

TIPE3	RAC1		Total
	High	Low	
High	45	22	67
Low	31	85	116
Total	76	107	183

*Note: r* = 0.305, *p* < 0.001.

## Data Availability

All the related data are available upon request by contacting with the corresponding author.
